# Insolation driven biomagnetic response to the Holocene Warm Period in semi-arid East Asia

**DOI:** 10.1038/srep08001

**Published:** 2015-01-23

**Authors:** Suzhen Liu, Chenglong Deng, Jule Xiao, Jinhua Li, Greig A. Paterson, Liao Chang, Liang Yi, Huafeng Qin, Yongxin Pan, Rixiang Zhu

**Affiliations:** 1 State Key Laboratory of Lithospheric Evolution, Institute of Geology and Geophysics, Chinese Academy of Sciences, Beijing 100029, China; 2 University of Chinese Academy of Sciences, Beijing 100049, China; 3 Key Laboratory of Cenozoic Geology and Environment, Institute of Geology and Geophysics, Chinese Academy of Sciences, Beijing 100029, China; 4 Key Laboratory of Earth and Planetary Physics, Institute of Geology and Geophysics, Chinese Academy of Sciences, Beijing 100029, China; 5 Research School of Earth Sciences, The Australian National University, Canberra, ACT 0200, Australia

## Abstract

The Holocene Warm Period (HWP) provides valuable insights into the climate system and biotic responses to environmental variability and thus serves as an excellent analogue for future global climate changes. Here we document, for the first time, that warm and wet HWP conditions were highly favourable for magnetofossil proliferation in the semi-arid Asian interior. The pronounced increase of magnetofossil concentrations at ~9.8 ka and decrease at ~5.9 ka in Dali Lake coincided respectively with the onset and termination of the HWP, and are respectively linked to increased nutrient supply due to postglacial warming and poor nutrition due to drying at ~6 ka in the Asian interior. The two-stage transition at ~7.7 ka correlates well with increased organic carbon in middle HWP and suggests that improved climate conditions, leading to high quality nutrient influx, fostered magnetofossil proliferation. Our findings represent an excellent lake record in which magnetofossil abundance is, through nutrient availability, controlled by insolation driven climate changes.

Magnetofossils, which are geologically preserved magnetic minerals produced by magnetotactic bacteria (MTB), have been proposed as distinctive environmental indicators^1^. Responses of MTB to palaeoenvironmental variations have been documented in various sedimentary settings, such as pelagic marine carbonates^2^,^3^ and haemipelagic marine^4^,^5^,^6^ and lake sediments^7^.

Hyperthermal events in geological times, such as Palaeocene-Eocene Thermal Maximum, Early Eocene Climate Optimum, Mid-Miocene Climate Optimum, and Holocene Warm Period (HWP), are believed to have profoundly changed global and/or regional hydrologic cycles, and thus significantly reshape sedimentary and biological processes in marine and terrestrial environments^8^,^9^. In particular, links between magnetofossil characteristics and hyperthermal events (mainly Palaeocene-Eocene Thermal Maximum) have been suggested based on several lines of evidence from ferromagnetic resonance, transmission electron microscope (TEM) and rock magnetism^3^,^4^,^5^,^6^,^9^,^10^.

The HWP is of particular interest to the palaeoclimate communities due to its significance for understanding the background of natural variability underlying anthropogenic climate change^11^. It is therefore essential to develop new comprehensive records of how the HWP impacted environmental and biological systems and to reconstruct the details of climate changes during this time period.

Here we report the first robust magnetofossil sequence through the HWP (9.8–5.9 ka) in lake sediments from the Asian interior. We investigated the magnetic properties of an 8.5-m Holocene lake sediment core from Dali Lake, Inner Mongolia, northern China (Fig. 1), in which the magnetic properties are controlled by biogenic and detrital magnetic minerals. We produce a 0.82 m magnetofossil sequence, which spans the 3.9-kyr HWP with a resolution of ~48 years. Our results document the first biomagnetic record of HWP climate variability at the northern limit of the East Asian summer monsoon. The biomagnetic response to the HWP is unambiguously demonstrated using a combination of magnetic measurements (Figs. 2,3,4) and TEM analyses (Fig. 5).

## Results

### Rock magnetism

The S-ratio, which is a composition-dependent parameter, is defined as the ratio of isothermal remanent magnetization acquired at −0.3 T (IRM_–0.3T_) to saturation IRM acquired at 1 T (IRM_1T_, hereinafter termed SIRM)^12^, has high values (generally above 0.9) throughout the studied core (Fig. 2a). This indicates the dominance of low-coercivity minerals (magnetite and/or maghaemite). Up-section, S-ratio generally decreases after the HWP, which indicates an increasing contribution of high-coercivity minerals (most likely aeolian haematite).

Low-field mass-specific magnetic susceptibility (χ), anhysteretic remanent magnetization (ARM) and SIRM, which are concentration-dependent parameters, have similar variations throughout the core (Fig. 2b–d). The HWP interval has notably high χ, ARM and SIRM values, which is due to the contribution from biogenic magnetite (i.e., magnetofossils), as indicated by analysis of first-order reversal curve (FORC)^13^,^14^,^15^,^16^ diagrams (Fig. 4) and TEM observations (Fig. 5). χ, ARM and SIRM for the pre-HWP sediments exhibit an up-section increasing trend, while the post-HWP sediments have nearly constant values, with the exception of isolated peaks. Importantly, the three parameters undergo a distinct two-stepped transition during the HWP. Specifically, early HWP (before 7.7 ka) values are generally low, but at 7.7 ka there is a noticeable increase, which is followed by a pronounced decrease in χ, ARM and SIRM at ~5.9 ka, the HWP termination.

Grain-size-dependent magnetic parameters χ_ARM_/χ (χ_ARM_ was determined from the ARM intensity, divided by the bias field strength), χ_ARM_/SIRM and SIRM/χ have similar variations throughout the core (Fig. 2e–g). The HWP sediments have higher values of these parameters than the pre- and post-HWP sediments, which indicates finer magnetic grain sizes in the HWP sediments compared with coarser grain sizes in the pre- and post-HWP sediments. A gradual up-section increase in χ_ARM_/χ, χ_ARM_/SIRM and SIRM/χ for the pre-HWP sediments is observed and indicates an up-section magnetic grain size decrease during the pre-HWP. Similar to the concentration-dependent parameters, there is also a rapid drop in these grain-size-dependent magnetic parameters at ~5.9 ka, which indicates a sudden decrease in fine-grained biogenic magnetite and an increase in coarse-grained detrital magnetite particles. A marked increase in χ_ARM_/χ and SIRM/χ is also discernable at ~7.7 ka, which suggests a significant increase in fine-grained ferrimagnetic biogenic magnetite particles.

Percentage of frequency-dependent magnetic susceptibility (χ_fd_%) has a nearly constantly low value of ~4% in the HWP sediments, but it has variable and higher values in the pre- and post-HWP sediments (Fig. 2h). This indicates an enrichment of superparamagnetic (SP) particles near the SD (single domain)/SP threshold size in the pre- and post-HWP sediments.

A bi-logarithmic plot of χ_ARM_/χ_fd_ (χ_fd_ is frequency-dependent magnetic susceptibility) versus χ_ARM_/χ (Fig. 3) aids in distinguishing between samples rich in biogenic magnetite and those rich in ferrimagnetic minerals derived from eroding soils^17^. Data for the HWP samples cluster in the upper right-hand corner of the plot and are characterized by high values of both χ_ARM_/χ_fd_ and χ_ARM_/χ, which indicates a dominance of SD magnetite (magnetofossils). In contrast, data for the pre- and post-HWP samples cluster in the lower left-hand corner of the plot. This is likely due to significant contributions from SP particles that have high χ and χ_fd_%, but low ARM values. The bi-logarithmic plot results, together with the correlation of concentration-dependant magnetic parameters (Supplementary Fig. 1), indicate different origins of magnetic minerals in the HWP samples compared with the pre- and post-HWP samples, which is confirmed by detailed rock magnetic and TEM analyses (Figs. 4,5).

### Mineral magnetic properties

Representative samples (DL550, 620, 670, 750, 790, and 850) from the core were selected for further magnetic analyses to identify the origin of the magnetic signal (Fig. 4, Supplementary Fig. 2 and Table 1). Hysteresis measurements generally indicate the dominance of low-coercivity ferrimagnetic minerals (Fig. 4a–c). Although coercivities of remanence (*B*_cr_) are comparable for all samples, the HWP samples generally have higher coercivities (*B*_c_) than those for pre- and post-HWP samples (e.g., ~20 to ~25 mT for the former, but ~10 mT for the latter; Supplementary Table 1). This difference may be due to the higher fraction of fine SP and coarser-grained magnetic particles within pre- and post-HWP samples compared with HWP samples, which will decrease the bulk *B*_c_, but affect *B*_cr_ less. This is confirmed by χ_fd_% values (Fig. 2h) and low-temperature magnetic measurements (Fig. 4d–f).

The sharp IRM decrease at ~100 K in SIRM_20K_2.5T_ curves (Fig. 4e) for sample DL670, known as the Verwey transition^18^, demonstrates the presence of magnetite. The observed Verwey transition temperatures (*T*_v_ = 95–110 K) are comparable with *T*_v_ for SD magnetite produced by modern MTB^19^,^20^, but are lower than for stoichiometric coarse-grained magnetite (≈120 K). In contrast, Verwey transition signals are not as strongly apparent for pre- and post-HWP samples (Fig. 4d,f), which can be explained by the presence of large amounts of SP magnetite or/and partial magnetite oxidation^21^.

Variable FORC diagrams are observed (Fig. 4g–k). For pre- and post-HWP samples, the FORC distribution peaks at *B*_c_ ≈ 9 mT (Fig. 4g,i). The inner contours close around the peak, while the outer contours remain open and intersect the y-axis, but do not diverge. This is indicative of pseudo-single domain (PSD) particles^13^,^16^. FORC distributions for HWP samples peak at *B*_c_ ≈ 38 mT with concentric contours (Fig. 4h) and horizontal elongation with little vertical spread, which indicates a dominance of high-coercivity non-interacting uniaxial SD particles. High-resolution FORC diagrams for HWP samples (Fig. 4j,k) yield a sharp central ridge that is characteristic of an assemblage of non-interacting SD particles. This is a strong indication of magnetofossils produced by MTB^14^,^15^.

### TEM analyses

To unambiguously detect magnetofossils within the HWP sediments, a magnetic extract was taken from sample DL670 and was analyzed with TEM techniques. Both TEM and HAADF (high angle annular dark field)-STEM (scanning TEM) observations reveal euhedral grains with diverse sizes and morphologies (Fig. 5). These particles range from ~20 nm to ~100 nm in size and have cubo-octahedral, prismatic, and bullet shapes, which are morphologically comparable with magnetites produced by modern MTB (e.g., ref. 22). HAADF-STEM-XEDS (X-ray energy dispersive spectra) mapping indicates that these euhedral particles are rich in iron and oxygen (Supplementary Fig. 3), which suggests that they are Fe oxides. High-resolution TEM (HRTEM) observations further indicate that these nanocrystals have the same crystal structure as magnetite. Overall, comprehensive TEM analyses provide strong morphological, chemical and structural evidence for a biogenic origin of magnetite within the HWP sample.

## Discussion

Combined rock magnetic and TEM analysis reveals that the HWP sediments are magnetically dominated by magnetofossils. Magnetofossils that survive in geological records benefit from both magnetosome production and preservation. Magnetofossil concentration in geological record, therefore, may depend mainly on magnetosome production^10^ or preservation^7^ or both^5^. In this study, however, dissolution of biogenic magnetite is not likely to be a major factor. The presence of large amounts of SP particles (Fig. 2h) and absence of iron sulphides (as indicated by the absence of sulphide oxidation during thermomagnetic analyses; Supplementary Fig. 2) in the post-HWP sediments suggest the absence of reductive dissolution in Dali Lake sediments. The relatively high abundance of magnetofossils in Dali Lake is, therefore, a result of MTB proliferation and biomineralization rather than being an artifact of preservation. It is a widely held view that MTB live in regions with strong vertical chemical gradients near the oxic-anoxic interface^23^. MTB populations cultured under a wide range of oxygenation conditions^24^ and magnetofossils found in sediments that were never anoxic^25^, however, indicate that such chemically stratified environments are not necessarily required for MTB population to flourish. Magnetosomes, due to their small particle size, tend to dissolve when buried under anoxic conditions^1^. In Dali Lake, MTB appear to have lived in a microaerobic environment and their magnetosomes were subsequently buried as magnetofossils in suboxic, but never anoxic conditions^2^.

Proliferation of MTB in Dali Lake sediments coincides with a period with warm and wet conditions due to increased insolation during the HWP (Fig. 6). High temperature and abundant precipitation during the HWP would have resulted in increased surface runoff and stronger chemical weathering. This would have enhanced catchment erosion and, therefore, increased the input of iron-rich basalt and terrestrial bio-matter into the lake. Increased surface runoff, evidenced by higher total organic carbon (TOC) and higher lake levels during the HWP^26^,^27^, would bring abundant nutrients including bioavailable iron and organic carbon into the lake for MTB biomineralization^2^. Therefore, magnetofossil abundance can be attributed to increased nutrient delivery into the lake in association with insolation-driven warm and wet HWP conditions.

The pronounced increase in ferrimagnetic mineral concentration and upward fining magnetic grain size at ~9.8 ka are caused by the transition from detrital magnetite derived from the catchment to biogenic magnetofossils produced within the lake. This increase corresponds to the onset of the HWP, which coincided with peak insolation at 65°N (ref. 28) (Fig. 6).

The rapid shift of magnetic minerals from magnetofossils to detrital magnetite at ~5.9 ka corresponds to the sudden termination of the HWP, which, in turn, is linked to the rapid insolation decrease at 65°N (ref. 28) (Fig. 6). Decreased surface runoff during the cold and dry post-HWP period would have resulted in a decreased nutrient supply, which would have led to a decline in MTB population. Therefore, the rapid shift from biogenic to detrital dominance of magnetic minerals in Dali Lake during 6–5.7 ka is probably related to drying at ~6 ka in the Asian interior^29^. The ~6 ka drying marks the abrupt termination of the HWP in central Inner Mongolia. This finding, combined with previous observations from the northwestern Pacific^29^, North Atlantic^30^, West Africa^31^,^32^,^33^, America^34^ and South China^35^, further suggests the global nature of this abrupt climate shift.

On the basis of highly magnetic HWP sediments, the two-step biomagnetic signal before and after ~7.7 ka correlates well with inferred changes in the source of water flowing into the lake. Previous studies of total organic and inorganic carbon concentrations in Dali Lake indicate that water input during the early Holocene originated mostly from snow/ice melt with less catchment erosion and land-derived organic matter^26^. For the late HWP, however, Dali Lake received water mostly from monsoonal precipitation. The warmer and wetter climate after ~7.7 ka would have brought more monsoonal rainfall, which have caused greater surface runoff from the catchment and allowed vegetation to thrive. Increased runoff would have brought higher quality nutrients into the lake. This further indicates that the two-stage biomagnetic behaviour (increase at ~7.7 ka) reflects a biomagnetic response to increased nutrient delivery.

To summarize, high-resolution rock magnetic analyses of the DL04 sediment core from Dali Lake, northern China, reveal that the HWP sediments are magnetically dominated by magnetofossils derived from MTB magnetosomes. The abundance of magnetofossils reflects conditions during the HWP when favourable climate and associated improved nutrient supply, such as bioavailable iron and organic carbon, enhanced the ability of MTB to biomineralize magnetite. In contrast, magnetic minerals in the pre- and post-HWP sediments mainly consist of detrital minerals from catchment erosion of bedrock and soils. Absence of biogenic magnetite in intervals that preceded and succeeded the HWP could be attributed to low nutrient flux under cold and dry conditions. The transition from detrital to biogenic magnetite at ~9.8 ka marked the onset of the HWP and is linked to postglacial warming, while the rapid shift from biogenic to detrital dominance of magnetite at ~5.9 ka marked the termination of the HWP and is linked to drying of the Asian interior at ~6 ka. Enhanced proliferation of biogenic magnetite at ~7.7 ka corresponds to the inferred turning point at which water input into Dali Lake transfers from colder snow/ice melt to warmer monsoonal precipitation at ~7.6 ka (ref. 26). The shift from snow/ice covered to more exposed terrain in the catchment area of Dali Lake is likely to have resulted in an increased nutrient flux due to direct erosion of the catchment area by monsoonal precipitation, which resulted in further proliferation of MTB. Our biomagnetic record correlates well with changes in summer insolation at high northern latitudes, which reflects the response of biotic systems in semi-arid lakes to insolation-driven climate changes through magnetic mineral production and deposition.

## Methods

### Geology, sampling and chronology

**Figure 1:**
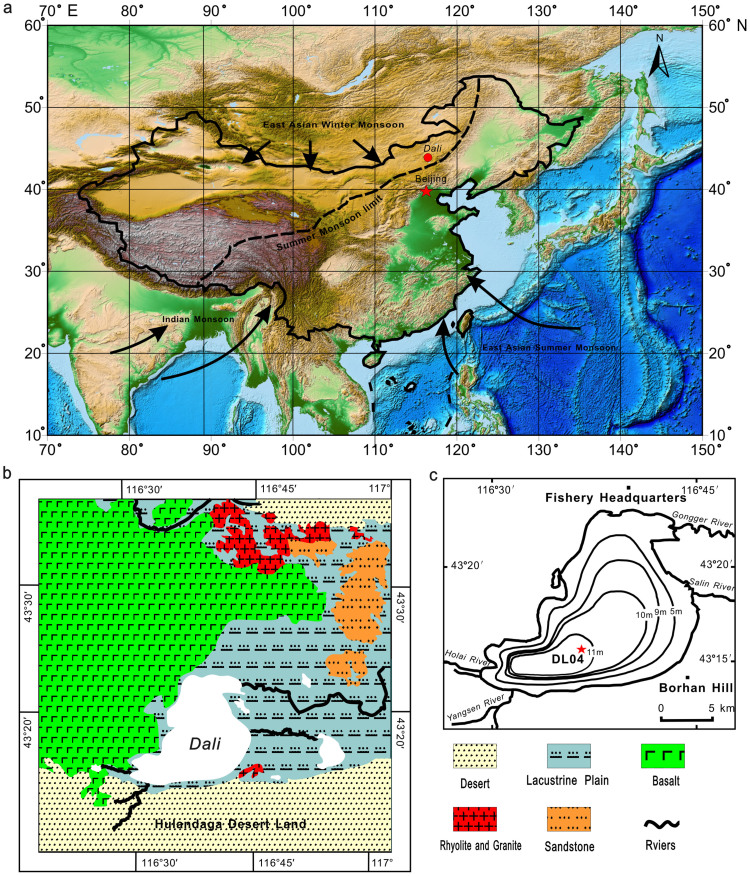
Maps of the setting of Dali Lake and the coring site. (a) Regional map with atmospheric circulation (black arrows): the East Asian summer monsoon, East Asian winter monsoon and Indian monsoon. Boundary (black dashed line): modern summer monsoon limit. (b) Geological setting of Dali Lake. (c) Bathymetric map of Dali Lake with the four main inflowing rivers. The red star represents the location of sediment core DL04. Part (a) is generated using the open and free software DIVA-GIS 7.5 (http://www.diva-gis.org/).

Dali Lake (43°13′–23′ N, 116°29′–45′ E), which is located in central Inner Mongolia at 1226 m.a.s.l., is an inland closed-basin lake structurally dammed by Pleistocene basalt (Fig. 1). This lake is located on the modern limit of the East Asian monsoon (Fig. 1a). Erosion in the surrounding catchment area is, therefore, likely to be influenced by monsoon variability. Pleistocene basaltic rocks surround the lake to the north and west. The E–W trending Hulandaga Desert Land lies to the south. Along the eastern shore are lacustrine plains (Fig. 1b). Because of its location in a semi-arid area with large evaporation and absence of draining rivers, Dali Lake is a soda brackish lake with salinity of up to 5.6‰ and pH of 9.6 (ref. 36). The lake has an area of 238 km^2^ and a maximum water depth of 11 m. The lake floor has a steep slope near the shore and an almost flat bottom toward the centre (Fig. 1c).

The studied DL04 sediment core (43°15.68′ N, 116°36.26′ E) was drilled at the depocenter, where there should be no significant water turnover. Drilling was performed in 2004 as described in ref. 26. Sediment cores were extracted to a depth beneath the lake floor of 12.57 m. The cored sediments are composed of greenish-grey to blackish-grey homogeneous silts and silty clays^26^. The core was split and cut on site at 1-cm intervals.

The chronology for core DL04 is based on linear interpolation between 15 accelerating mass spectra (AMS)-radiocarbon dates on bulk samples (Supplementary Table 2). The 12.57-m core covers the last 17.2 kyr. The upper 8.5 m of the DL04 core, which covers the last 11.5 kyr (the Holocene), was used in this study. A total of 850 unoriented sediment samples were obtained from this interval.

**Figure 2:**
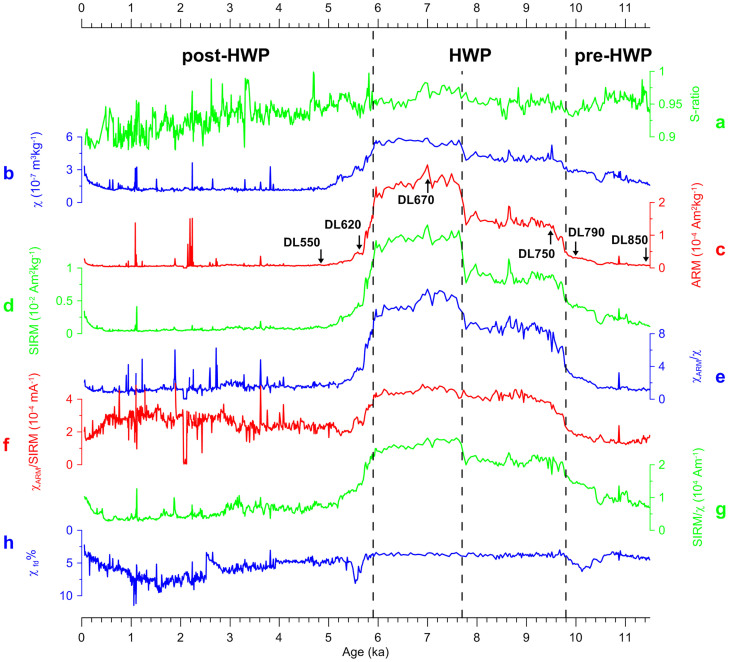
Time series of magnetic parameters. (a) S-ratio. (b) χ. (c) ARM. (d) SIRM. (e) χARM/χ. (f) χARM/SIRM. (g) SIRM/χ. (h) χfd%. The sequence is divided into three units (pre-HWP, HWP and post-HWP), see text for details. Positions of representative samples are shown in (c).

**Figure 3:**
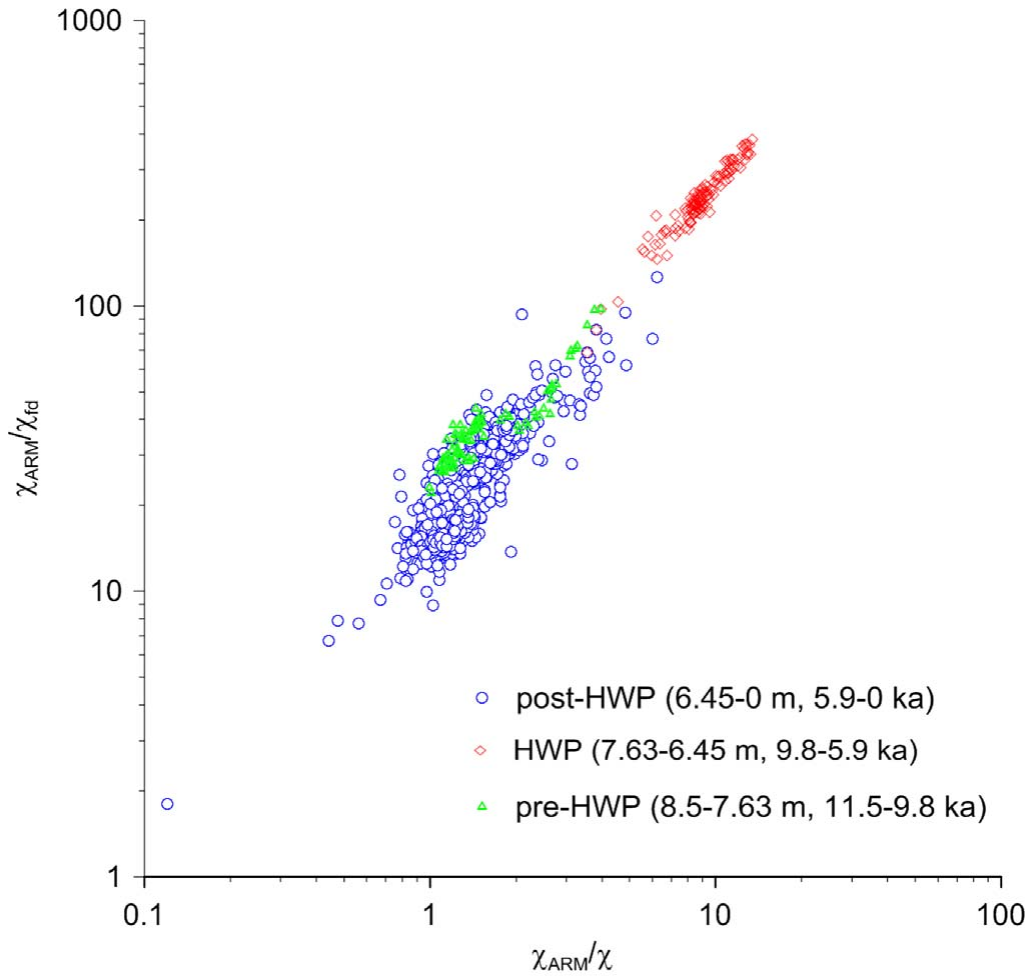
Bi-logarithmic plot of χ_ARM_/χ_fd_ versus χ_ARM_/χ. Samples clustered in the upper right-hand-side of the plot are indicative of biogenic magnetite, while samples in the lower left-hand-side are indicative of detrital input from soil erosion^17^.

Based on variations of magnetic parameters and the chronology from radiocarbon dating, we divide the sequence into three units: the pre-HWP unit (8.50–7.63 m, 11.5–9.8 ka), the HWP unit (7.63–6.45 m, 9.8–5.9 ka), and the post-HWP unit (6.45–0 m, 5.9–0 ka) (Fig. 2). The HWP sediments are dominated by biogenic magnetite, and the pre- and post-HWP sediments, by detrital magnetite.

### Magnetic measurements

Magnetic measurements were carried out on all the 850 unoriented bulk sediment samples. The samples were first dried in vacuum and placed into standard 8 cm^3^ palaeomagnetic cubes. χ was measured using an AGICO MFK1-FA Multi-Frequency Kappabridge magnetic-susceptibility meter at frequencies of 976 Hz and 15616 Hz. Two measures of frequency-dependent magnetic susceptibility (χ_fd_, defined as χ_976Hz_ – χ_15616Hz_, and χ_fd_%, defined as (χ_976Hz_ – χ_15616Hz_)/χ_976Hz_ × 100%) were calculated from these measurements. χ_fd_% was used to identify particles close to the SP/SD transition. ARM was imparted using a peak alternating field (AF) of 100 mT and a direct bias field of 0.05 mT using a 2-G Enterprises SQUID magnetometer with inline AF coil. IRM was induced with a 2-G Enterprises model 660 pulse magnetizer successively in pulsed fields of 1 T and –0.3 T. The S-ratio^12^ was determined to estimate the relative contributions of low-coercivity magnetic minerals (e.g., magnetite, maghaemite, and greigite) versus high-coercivity magnetic minerals (e.g., haematite and goethite). All remanence measurements were measured using the 2-G magnetometer.

### Magnetic properties of representative samples

Low-temperature magnetic measurements were performed on a Quantum Design Magnetic Property Measurement System (MPMS-XL). Samples were first cooled from 300 K to 20 K in zero field (ZFC). At 20 K an SIRM was imparted with a 2.5 T field (hereafter termed SIRM_20K_2.5T_) and the remanence was measured during zero field warming to 300 K. *T*_v_ of magnetite is defined as the temperature of the minimum in the first derivative of the ZFC curve.

**Figure 4:**
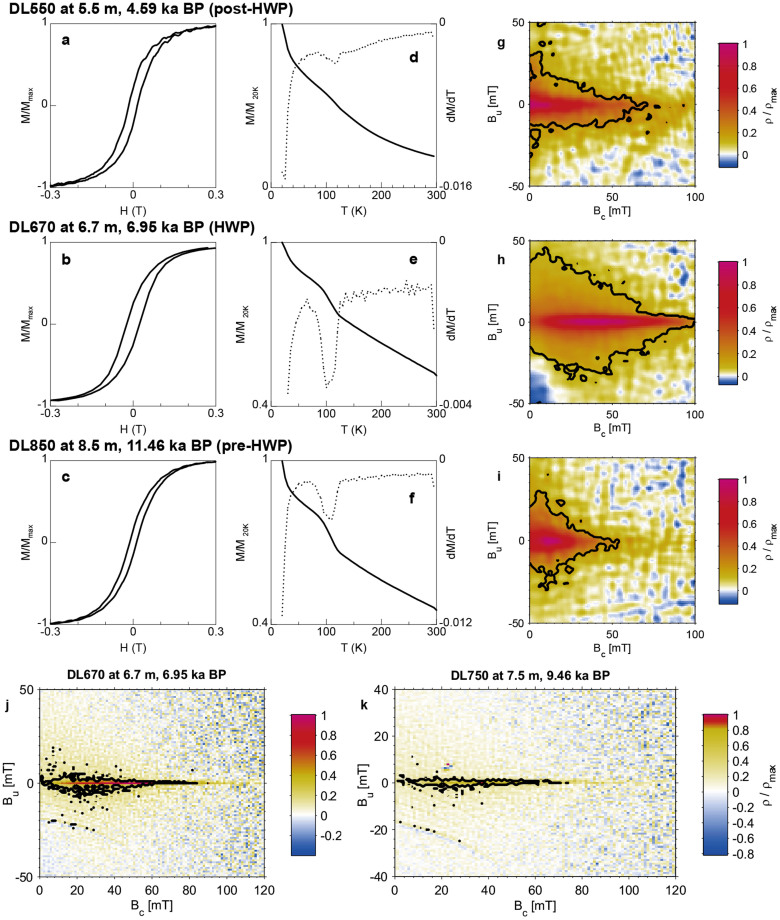
Representative rock magnetic results. Samples DL670 and DL750 represent the HWP sediments dominated by biogenic magnetite, while samples DL550 and DL850 represent sediments magnetically dominated by detrital magnetite. (a–c) Hysteresis loops after paramagnetic slope correction. To get higher signal-to-noise ratios, three measurements were repeated and averaged. (d–f) Solid lines represent thermal demagnetization curves of a low-temperature IRM imparted at 20 K in a 2.5 T field after ZFC treatment. Moments are normalized by the value at 20 K. The dotted lines represent the first derivative, dM/dT, of the warming curve after ZFC treatment. (g–i) Low-resolution FORC diagrams, measured with δB = 0.876 mT and calculated with SF = 4. (j–k) High-resolution FORC diagrams of the HWP samples, measured with δB = 0.4 mT and calculated with SF = 4. The thick contour lines indicate the regions of the FORC distribution that are significant at the 0.05 level^37^. The diagrams in (j–k) are averages of three FORC runs.

**Figure 5:**
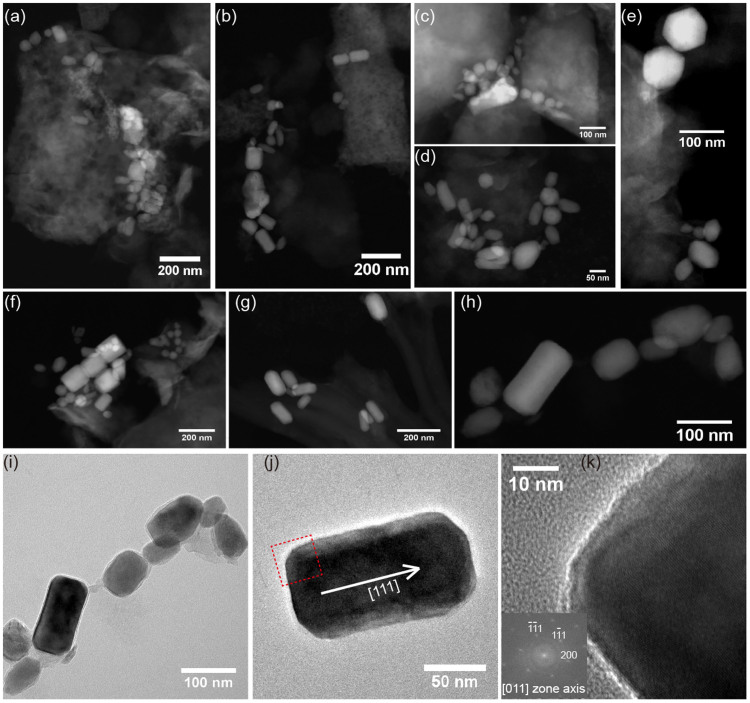
TEM analyses of magnetofossils from the HWP sediments (DL670). (a–h) HAADF-STEM images of magnetofossils. HAADF-STEM allows Z-contrast imaging, which is suitable for magnetofossil identification based on morphological features and contrast with the surrounding matrix. (i) Bright-field TEM image of magnetofossils in (h). (j) HRTEM image of a typical prismatic magnetite recorded from [011] zone axis. (k) Close-up image of the area indicated by the red dashed square in (j). The corresponding fast Fourier transform (FFT) pattern is indexed and shown in the inset. HRTEM imaging and the FFT pattern indicate that this particle is elongated along the [111] direction of magnetite, which is consistent with cubo-octahedral magnetosomes^22^.

**Figure 6:**
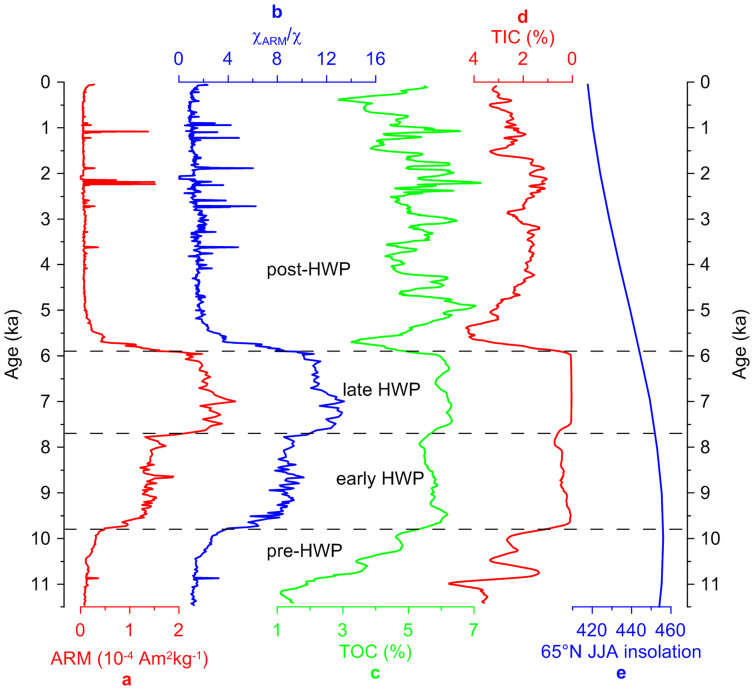
Comparison of magnetic mineral concentration (ARM) and magnetic grain size (χ_ARM_/χ) with total organic carbon (TOC), total inorganic carbon (TIC) and the summer (June, July, August) average insolation computed for 65°N (ref. 28). High TOC and low TIC reflect high lake level (data from ref. 26). Note the reversed scale in (d). The black dashed lines separate the different time intervals (pre-, early, late and post-HWP).

Hysteresis loops, IRM acquisition, back-field demagnetization of SIRM, and FORCs (Fig. 4) were measured on a Princeton Measurements Corporation MicroMag 3900 vibrating sample magnetometer (VSM) up to a maximum field of 1.5 T. FORC diagrams were produced using the FORCme software^37^. Two selected samples with FORC distributions typical of non-interacting SD assemblages of biogenic magnetite (Fig. 4j,k) were further analyzed following the high-resolution FORC protocol described in ref. 14. To improve the signal-to-noise ratio of FORC diagrams, three FORC measurements were repeated and stacked. All diagrams were constructed using a smoothing factor of 4.

### TEM analysis

For TEM observations, magnetic extracts were obtained from bulk sediments by stirring the mortared sediment in a small volume of distilled water before thoroughly dispersing by ultrasonication. The magnetic particles were then extracted with a magnetic finger. To concentrate and purify the magnetic components, the extraction procedure was repeated at least five times. TEM experiments were carried out on a JEOL-2100F microscope operating at 200 kV, equipped with a field emission gun, an ultra-high-resolution (UHR) pole piece, a Gatan energy filter GIF 200, a JEOL detector with an ultrathin window allowing detection of light elements, and a STEM device, which allows Z-contrast imaging in HAADF mode. Compositional mapping was acquired by performing XEDS analysis in the STEM mode.

## Author Contributions

C.D., J.X., J.L., Y.P. and R.Z. designed the study. J.X. collected samples. S.L., J.L. and H.Q. conducted experiments. S.L., J.L., G.A.P., L.C., L.Y. and C.D. wrote the paper. All authors contributed to data interpretation and provided significant input to the final manuscript.

## Supplementary Material

Supplementary Information supplementary information to Insolation driven biomagnetic response to the Holocene Warm Period in semi-arid East Asia
