# Supplementary material for: Insolation driven biomagnetic response to the Holocene Warm Period in semi-arid East Asia
Source: Sci Rep. 2015 Jan 23;5:8001. doi: 10.1038/srep08001 (PMC4303925; doi:10.1038/srep08001)
Supplement: Supplementary Information — to Insolation driven biomagnetic response to the Holocene Warm Period in semi-arid East Asia [file srep08001-s1.doc]

**SUPPLEMENTARY INFORMATION**

**Insolation driven biomagnetic response to the Holocene Warm Period in semi-arid East Asia**

Suzhen Liu^1,2^, Chenglong Deng^1^,*, Jule Xiao^3^, Jinhua Li^4^, Greig A. Paterson^4^, Liao Chang^5^, Liang Yi^1^, Huafeng Qin^1^, Yongxin Pan^4^ & Rixiang Zhu^1^

**Supplementary Figures**

**
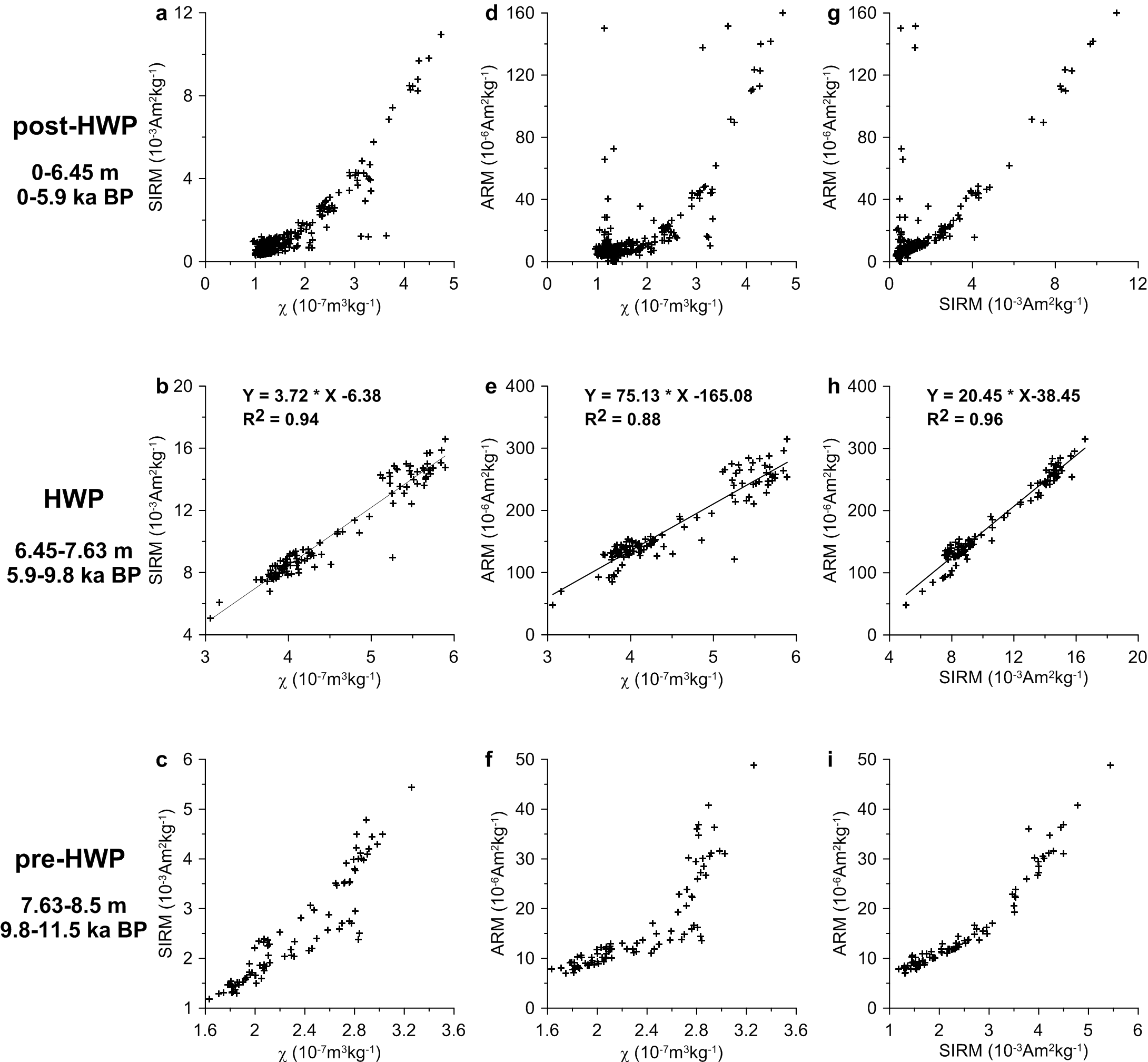
**

**Supplementary Figure S1 | Correlation plots of magnetic parameters from samples from the pre-HWP, HWP and post-HWP sediments of Dali Lake.** (a–c) SIRM versus χ. (d–f) ARM versus χ. (g–i) ARM versus SIRM. Statistically significant correlations between the plotted parameters (b, e, h, R^2^=0.94, 0.88, 0.96) for the HWP samples demonstrate the highly uniform grain size distributions, as would be expected from biogenic magnetite. For other parts of the core (a, c, d, f, g, i), there is no such obvious linear correlation, which indicates a wider magnetic grain size distribution indicative of detrital influx.


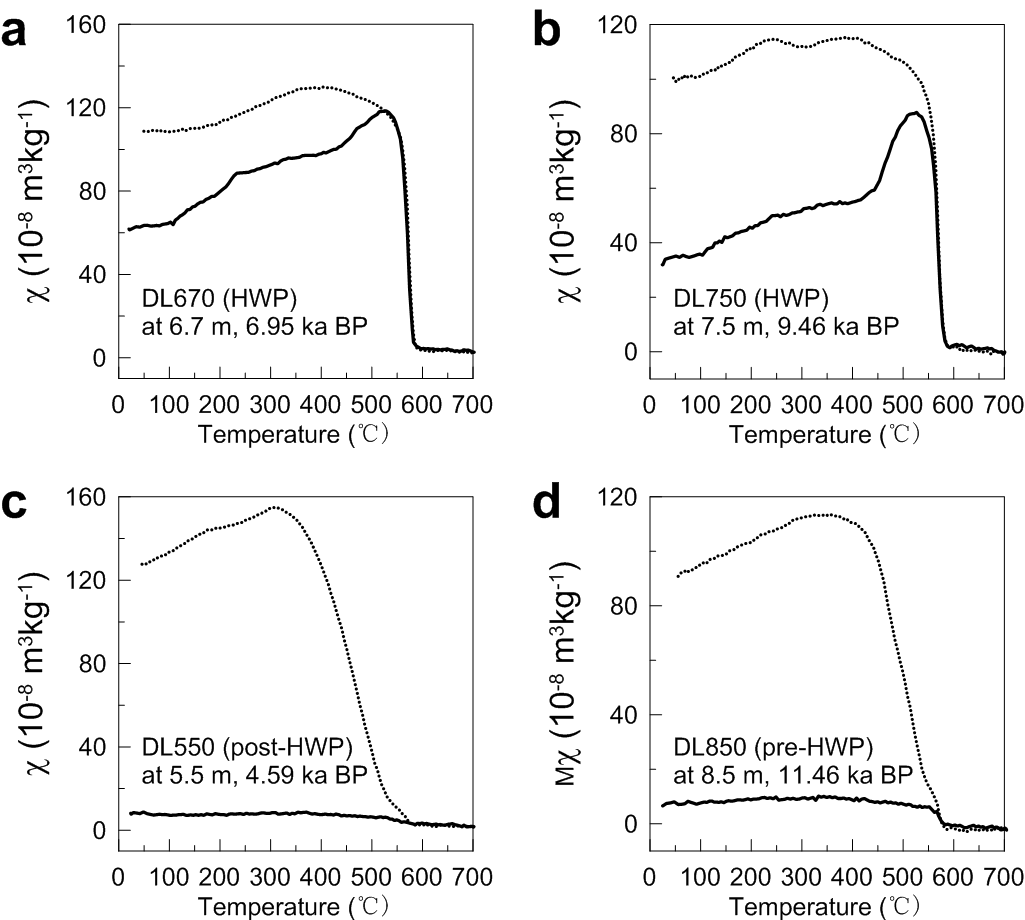


**Supplementary Figure S2 | Temperature dependence of magnetic susceptibility (χ-T curves) for representative samples.** (a–b) Samples during the HWP. (c) Sample from the post-HWP sediments. (d) Sample from the pre-HWP sediments. The sharp susceptibility decreases at 570–580ºC and nearly reversible trends at high temperature for the HWP samples (a–b) are consistent with the low-temperature magnetic and FORC results that indicate biogenic SD magnetite dominates the HWP sediments. For the pre- and post-HWP samples, the nearly temperature-independent nature of low-field susceptibility below the Curie point of magnetite indicates that detrital coarse-grained (large pseudo-single domain or multidomain-like) magnetite is the major contributor to the magnetic susceptibility^1^. The large susceptibility increase after heating of the pre- and post-HWP sediments is likely due to the formation of magnetite from iron-bearing clay minerals during heating or transformation of highly oxidized iron-oxides to magnetite in an argon atmosphere^2^.

**
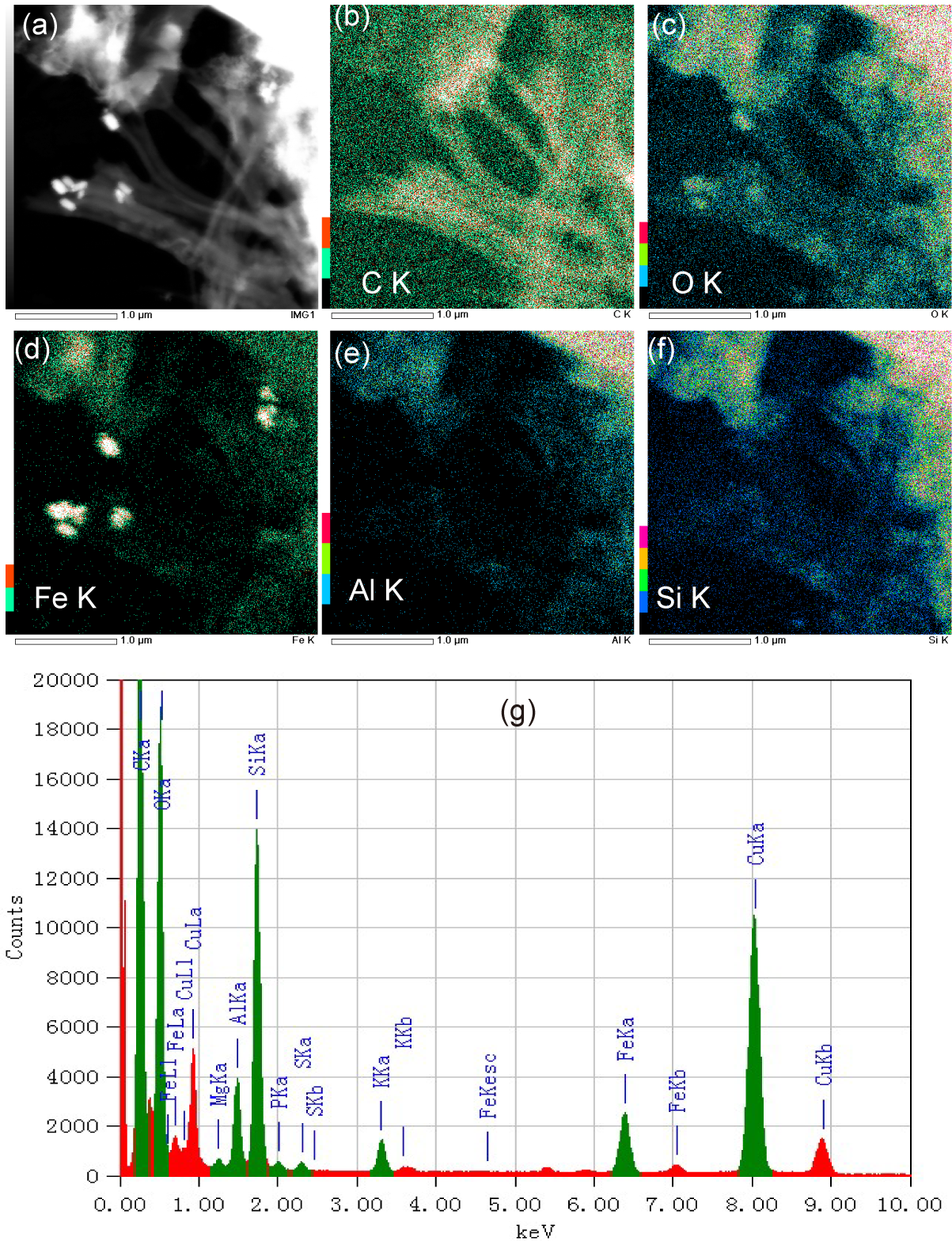
**

**Supplementary Figure S3 | Chemical characterization of magnetofossils.** (a) HAADF-STEM image of several prismatic magnetofossils associated with some fibres. (b–f) HAADF-STEM-XEDS maps of the spatial distribution of C, O, Fe, Al, and Si. (g) X-ray energy dispersive spectrum.

**Supplementary Tables**

**Supplementary Table S1 | Summary of magnetic hysteresis parameters of representative samples**

The samples are shown according to depth in the core. Samples DL670 and DL750 are the HWP sediments that are dominated by biogenic magnetofossils. Samples DL850 and DL550 are pre- and post-HWP sediments, respectively, and contain mainly detrital magnetic minerals. Samples DL620 and DL790 represent sediments from transitional intervals.

| **Sample ID** | **Depth**  **(m)** | **Age**  **(ka)** | ***B*_c_**^a^  **(mT)** | ***B*_cr_**^b^  **(mT)** | **M_rs_/M_s_** | ***B*_cr_/*B*_c_** | **σ_hys_**^c^ | **S-ratio** |
| --- | --- | --- | --- | --- | --- | --- | --- | --- |
| DL550 | 5.5 | 4.59 | 14.7 | 48.5 | 0.21 | 3.30 | 0.02936 | 0.93 |
| DL620 | 6.2 | 5.49 | 13.0 | 38.7 | 0.15 | 2.98 | -0.01930 | 0.94 |
| DL670 | 6.7 | 6.95 | 23.2 | 49.8 | 0.25 | 2.15 | -0.11481 | 0.98 |
| DL750 | 7.5 | 9.46 | 21.8 | 50.5 | 0.23 | 2.32 | -0.66180 | 0.95 |
| DL790 | 7.9 | 10.52 | 10.8 | 33.0 | 0.12 | 3.06 | -0.11085 | 0.95 |
| DL850 | 8.5 | 11.46 | 12.0 | 41.8 | 0.14 | 3.48 | -0.05888 | 0.94 |

^a^Coercivity.

^b^Coercivity of remanence.

^c^Hysteresis shape parameter calculated from a hysteresis loop^3^, with σ_hys_ <0 (σ_hys_ >0) for wasp-waisted loops (potbellied loops), respectively.

**Supplementary Table S2 |Radiocarbon dating results**

| **Lab number** | **Depth (cm)** | **δ^13^C (‰)** | **AMS ^14^C age**  **(^14^C yr BP)** | **Calibrated ^14^C age (2σ)**  **(cal yr BP)** |
| --- | --- | --- | --- | --- |
| PLD–4564 | 0.5 | –28.19 ± 0.11 | 472 ± 23 | 74–31 |
| PLD–4565 | 99.5 | –25.47 ± 0.11 | 1463 ± 24 | 964–891 |
| PLD–6250 | 150 | –28.05 ± 0.23 | 1629 ± 22 | 1172–1045 |
| PLD–4567 | 200 | –24.65 ± 0.12 | 1948 ± 25 | 1414–1299 |
| PLD–12450 | 299 | –23.24 ± 0.14 | 2652 ± 22 | 2318–2115 |
| PLD–12453 | 350 | –24.60 ± 0.17 | 2879 ± 23 | 2503–2347 |
| PLD–12456 | 449 | –25.36 ± 0.19 | 3561 ± 24 | 3381–3240 |
| PLD–12459 | 500 | –27.80 ± 0.10 | 4113 ± 25 | 4013–3866 |
| PLD–6706 | 550 | –23.66 ± 0.13 | 4562 ± 21 | 4655–4517 |
| PLD–4572 | 600 | –27.24 ± 0.12 | 4947 ± 28 | 5293–5032 |
| PLD–6255 | 650 | –25.58 ± 0.18 | 5709 ± 26 | 6030–5917 |
| PLD–12466 | 699 | –30.69 ± 0.18 | 8014 ± 29 | 8417–8308 |
| PLD–12469 | 750 | –27.98 ± 0.17 | 8881 ± 30 | 9524–9398 |
| PLD–12470 | 799 | –31.56 ± 0.11 | 9969 ± 32 | 10870–10654 |
| PLD–12472 | 849 | –30.84 ± 0.19 | 10464 ± 37 | 11640–11268 |

**References**

1 Deng, C. L., Shaw, J., Liu, Q. S., Pan, Y. X. & Zhu, R. X. Mineral magnetic variation of the Jingbian loess/paleosol sequence in the northern Loess Plateau of China: Implications for Quaternary development of Asian aridification and cooling. *Earth Planet. Sci. Lett.* **241**, 248-259, doi:10.1016/j.epsl.2005.10.020 (2006).

2 Deng, C., Zhu, R., Jackson, M. J., Verosub, K. L. & Singer, M. J. Variability of the temperature-dependent susceptibility of the Holocene eolian deposits in the Chinese loess plateau: A pedogenesis indicator. *Phys. Chem. Earth (A)* **26**, 873-878, doi:10.1016/S1464-1895(01)00135-1 (2001).

3 Fabian, K. Some additional parameters to estimate domain state from isothermal magnetization measurements. *Earth Planet. Sci. Lett.* **213**, 337-345, doi:10.1016/S0012-821X(03)00329-7 (2003).
